# Combining hippocampal volume metrics to better understand Alzheimer’s disease progression in at-risk individuals

**DOI:** 10.1038/s41598-019-42632-w

**Published:** 2019-05-16

**Authors:** K. McRae-McKee, S. Evans, C. Hadjichrysanthou, M. M. Wong, F. de Wolf, R. M. Anderson

**Affiliations:** 1 Department of Infectious Disease Epidemiology, School of Public Health, Faculty of Medicine, Imperial College London, St Mary’s Campus, Norfolk Place, W2 1PG London, United Kingdom; 2 Janssen Prevention Center, Archimedesweg 4, 2333 CN Leiden, The Netherlands

**Keywords:** Predictive markers, Alzheimer's disease

## Abstract

To date nearly all clinical trials of Alzheimer’s disease (AD) therapies have failed. These failures are, at least in part, attributable to poor endpoint choice and to inadequate recruitment criteria. Recently, focus has shifted to targeting at-risk populations in the preclinical stages of AD thus improved predictive markers for identifying individuals likely to progress to AD are crucial to help inform the sample of individuals to be recruited into clinical trials. We focus on hippocampal volume (HV) and assess the added benefit of combining HV and rate of hippocampal atrophy over time in relation to disease progression. Following the cross-validation of previously published estimates of the predictive value of HV, we consider a series of combinations of HV metrics and show that a combination of HV and rate of hippocampal atrophy characterises disease progression better than either measure individually. Furthermore, we demonstrate that the risk of disease progression associated with HV metrics does not differ significantly between clinical states. HV and rate of hippocampal atrophy should therefore be used in tandem when describing AD progression in at-risk individuals. Analyses also suggest that the effects of HV metrics are constant across the continuum of the early stages of the disease.

## Introduction

As of 2012, phase III clinical trials of possible Alzheimer’s disease (AD) therapies had a failure rate of 99.6%^1^. One recent example was that of a beta-secretase (BACE) inhibitor, designed by Merck, targeted at patients with mild-to-moderate AD^2,3^. A number of factors are thought to have contributed to these failures, including sub-optimal patient-selection approaches (e.g. the targeting of individuals already diagnosed with AD as opposed to those in the preclinical stage of the disease), large variation both between and within individuals, poor endpoint choice, and potential measurement error in outcome and explanatory measures (biomarkers, imaging markers and neuropsychological assessments)^4,5^. Another reason may be the inclusion of participants without evidence of amyloid pathology^6^ thereby including individuals that are less likely to decline clinically over the duration of the trial. Clinical trials are beginning to target at-risk populations in the preclinical stages of AD with the belief that this may increase the chance of either preventing or delaying the onset of AD^7^. A draft guidance report from the Food and Drug Administration defines four classifications of individuals through the stages of showing no clinical symptoms to overt dementia based on pathophysiologic, cognitive and functional changes to help identify individuals in the preclinical stages of the disease^8^. This reinforces the need for predictive markers to help inform the sample of individuals that present with little or no symptoms of AD at the beginning of the trial that is more likely to develop AD over the course of the study.

Reduced hippocampal volume (HV) has been shown to have a strong association with progression to AD^9^ and changes significantly over time in individuals with late mild cognitive impairment^5^. Additionally, HV tends to be recorded more consistently across epidemiological studies, both cross-sectionally and longitudinally, relative to other markers. For example, individuals that participate in the Alzheimer’s Disease Neuroimaging Initiative (ADNI) have on average 3.93 HV measurements taken with 1,485 cross-sectional measurements of HV collected at baseline.

Studies have investigated the predictive value of cross-sectional HV measures such as raw values, normalised volumes and rates of hippocampal atrophy, typically by estimating hazard ratios and/or the area under the curve (AUC)^10–14^. A small number of studies^15,16^ considered the association between rates of hippocampal atrophy and AD and found that greater hippocampal atrophy was associated with mild cognitive impairment (MCI) and AD diagnoses and that this association became stronger over time, albeit limited to 12 months from baseline. However, no published studies have been identified that have investigated the effect of HV on disease progression by utilising extensive, long term longitudinal MRI data, such as that available in ADNI. Furthermore, with the exception of Rana and colleagues^12^ through a study in which cross-sectional HV and rates of hippocampal atrophy were used to distinguish between diseased and non-diseased individuals, there is a gap in the literature when it comes to using a combination of absolute HV and rate of hippocampal atrophy to better understand the progression of AD over longer periods of time. The present study addresses this gap using a sample of individuals at high risk of progressing as defined by abnormal levels of Aβ_42_ markers at baseline.

The aims of the study are three-fold: (1) to cross-validate previously reported predictive scores related to HV markers, (2) to identify the best combination of HV metrics in terms of understanding disease progression over time and (3) to investigate if different HV metrics should be used to maximise the precision in the estimation of the likelihood of progressing from different clinical states (i.e. cognitively normal (CN) and MCI). In other words, should the rate of hippocampal atrophy be used to predict disease progression specifically from CN, or from MCI, or both?

Lastly, we bring together the findings of this study to discuss the manner in which the MRI methods described in this paper can be used in the patient screening phase of clinical trials of potential AD therapies to help increase power, as well as to inform the simulation of clinical trial design^4,17,18^ prior to implementation.

## Methods

### ADNI data

The data used in this study were acquired from the ADNI database (http://adni.loni.usc.edu). The data used was downloaded on October 31st, 2016.

Measurements on a large number of clinical, biochemical and imaging markers are collected in ADNI with differing levels of longitudinal coverage for individual patients. The distributions by number of measurements by individual of a set of markers recorded in ADNI can be found in Table 1.

**Table 1:** The number of measurements recorded per marker per individual in ADNI irrespective of visit number.

	Number of measurements per individual						
	0	1	2	3	4	5+	Mean (SD)
HV	53	186	195	253	347	696	3.93 (2.00)
FDG	328	587	468	44	86	217	1.93 (1.92)
AV45	642	384	416	276	12	0	1.21 (1.13)
PIB	1,627	21	46	35	1	0	0.13 (0.54)
Aβ_42_	308	1,235	39	63	44	41	0.57 (1.17)
t-tau	328	1,213	39	69	42	39	0.55 (1.16)

Volumetric measurements, e.g. HV, are the most widely recorded among structural MRI, PET imaging and CSF markers across all visits. Fluoro-2-deoxy-D-glucose (FDG) and Florbetapir (AV45) PET markers are the next best in terms of overall data collection within individuals however not as consistently recorded over time in general with pre-defined influxes of data collection e.g. at 24-month and 48-month visits. CSF markers of Aβ_42_ and total-tau (t-tau) tend to be collected annually although not as often within individuals compared with volumetric measurements.

In addition to the quantity of measurements at the population level, analysis of the change in markers requires the collection of repeated measures within individuals over time. On average, two more HV measurements are collected per individual with 1,296 individuals having three or more.

MRI data were collected using a standardised protocol, which has been summarised in several publications^19,20^. In summary, all subjects had two T1-weighted scans at 1.5T with the scan of higher quality being selected by the ADNI MRI quality control centre at the Mayo Clinic (Rochester, MN, USA). Scans were collected using a sagittal volumetric magnetization-prepared rapid gradient echo (3D MP-RAGE) sequence having a number of fixed parameters and processed using FreeSurfer versions 4.3 (ADNI1) and 5.1 (ADNI2 and ADNIGO). Further detail relating to the ADNI MRI protocols can be found at http://adni.loni.usc.edu/data-samples/mri.

### Study participants

The sample of individuals considered in this study includes those enrolled in the ADNI study with two or more measures of HV and complete data recorded for age, gender, education, number of apolipoprotein E (APOE) epsilon 4 (ε4) alleles and clinical diagnosis at each visit (CN, MCI or AD). The sample was further restricted to exclude individuals diagnosed with AD at baseline and only included CN and MCI individuals that were identified as being amyloid positive and therefore at higher risk of developing AD (see methods section for description of amyloid classification). The implementation of this inclusion criteria meant that 561 out of 1730 individuals were eligible within the current study. Two volumetric measurements were required for each brain region, as hippocampal atrophy requires a reference point for calculation purposes. Detailed characteristics of the overall sample can be found in Table 2.

**Table 2:** Characteristics of the sample at baseline (n = 561).

		CN	MCI
Number of individuals		160	401
Number of visits	Mean (SD)	4.79 (1.97)	5.58 (1.93)
Time between visits (years)	Mean (SD)	0.89 (0.47)	0.76 (0.33)
Age at baseline (years)*	Mean (SD)	74.56 (6.31)	73.07 (6.96)
Gender*	Male (%)	43.75	58.35
Education (years)	>12 (%)	88.75	82.54
APOE ε4 genotype*	Negative (%)	56.88	35.41
	Hetero- (%)	38.12	49.63
	Homo- (%)	5.00	14.96

*Significant difference (p < 0.05) between CN and MCI groups.

Events within the predictive and time-to-event analyses were defined as conversion from MCI diagnosis at 6-month visit to AD and was limited to individuals with HV recorded both at baseline and at 6 months. Therefore, in combination with the criteria above, a subset of 284 individuals that were diagnosed as MCI after 6 months, had HV recorded both at baseline and at 6 months and had at least one diagnosed visit after 6 months form the sample for these analyses.

Subsequent analyses, which consider the iterative progression towards AD over time, allow for the consideration of individuals diagnosed as CN or MCI at baseline but exclude visits at which individuals are diagnosed with AD. For example, once individuals progress to AD, their subsequent visit is ignored. By definition, this includes baseline visits at which an individual has already been diagnosed with AD, hence the exclusion of these individuals. In summary, all 561 individuals contributed towards this analysis.

### Research involving human participants and/or animals

#### Ethical approval (humans)

As per ADNI protocols, all procedures performed in studies involving human participants were in accordance with the ethical standards of the institutional and/or national research committee and with the 1964 Helsinki declaration and its later amendments or comparable ethical standards. More details can be found at adni.loni.usc.edu. (This article does not contain any studies with human participants performed by any of the authors).

#### Ethical approval (animals)

This article does not contain any studies with animals performed by any of the authors.

### Protocol approval, ethics approval and consent to participate

The study was approved by the Institutional Review Boards of all the participating institutions and informed written consent was obtained from all participants at each site. One such institution is the Office for the Protection of Research Subjects at the University of Southern California. More details can be found at adni.loni.usc.edu.

## Measures

### Demographics

Age, education, gender and APOE ε4 genotype were not primary factors of interest but were considered as potential confounders and were therefore accounted for in all analyses. Age was considered as continuous (in years) and represented an individual’s age at the visit of interest. Education was dichotomised such that individuals were grouped as having either greater than (>) or less than or equal to (≤) 12 years of education. APOE ε4 genotype was considered as a factor with levels “negative”, “heterozygous” and “homozygous”. Furthermore, longitudinal models accounted for time between visits and diagnosis at previous visit.

### Amyloid classification

The sample in the present study was restricted to individuals that showed evidence of amyloid positivity defined as abnormal levels of Aβ_42_ in CSF or abnormal levels of amyloid plaques evidenced by AV45 PET, or both. A threshold of 192 pg/mL was used to dichotomise values of Aβ_42_ in CSF^21^ such that values below the threshold were considered to be abnormal. In the case of AV45 PET, the SUVR threshold was set at 1.08 and was defined such that it would classify 90% of individuals diagnosed as AD at baseline as abnormal. The details of this method are described elsewhere^22^.

### Markers of AD

Several metrics that quantify the levels of an individual’s absolute HV or rate of hippocampal atrophy were computed for each time step: HV adjusted for ICV using the residual^22^ and ratio^23^ methods, as well as the annualised rate of hippocampal atrophy. Two normalisation methods were considered (residual and ratio) to evaluate their respective predictive potential and carry forward and evaluate the metric with the higher predictive value within the longitudinal analysis.

HV residuals (HV_res_) were calculated as the difference between the raw HV values observed in the data and the fitted HV values from a linear regression of HV regressed on ICV in the sample^22^ as shown in Eq. 1.

1 $$H{V}_{res,j}=H{V}_{raw,j}-H{V}_{fitted,j},$$

where $j=\{0,1,\ldots ,N\}$ represents visit number. HV_res_ can be interpreted as the difference between an individual’s recorded HV and their expected HV based on their ICV, whereby negative values indicate a smaller HV than expected. HV_res_ can also be interpreted in the same way as normalised HV measures^24^.

An alternative method for the adjustment of HV relative to ICV is to simply compute the ratio between the two, HV_ratio_, as per Eq. 2,^23^,^25^.

2 $$H{V}_{ratio}=H{V}_{raw}/IC{V}_{raw}.$$

The rate of hippocampal atrophy per year (HV_rate_) was defined as the rate of change in HV measured at visit *j* relative to the HV measured independently at visit *j-*1, as per Eq. 3.

3 $$H{V}_{rate,j}=\frac{H{V}_{raw,j}-H{V}_{raw,j-1}}{{t}_{j}-{t}_{j-1}},$$

where, *t*_*j*_ represents the time of follow up visit *j* in years. Negative values for HV_rate_ indicate HV atrophy relative to the previous visit.

### Statistical Analysis

Statistical comparisons of the demographics and HV metrics between individuals (Table 2) were made using Chi-squared tests and independent samples t-tests for categorical and continuous variables, respectively.

Comparisons between the predictive value and risk associations of HV metrics were made using two methods: Receiver Operating Characteristic (ROC) curves and Cox proportional hazards regression. ROC curves are typically used to evaluate the ability of a measure in distinguishing between two diagnostic groups (AD progressors and non-progressors in this study). Cox regression is a time-to-event/survival analysis technique which estimates the effect of one or more covariates on the time to a well-defined event while accounting for potential confounders. In this study, proportional hazard models were adjusted for age, gender, education (dichotomised; >12 years and ≤12 years) and APOE ε4 genotype (negative, heterozygous, homozygous) and HV metrics were grouped into tertiles with the first tertile serving as the reference group. Increasing tertiles were coded to represent increasing risk of progression.

Longitudinal modelling of HV and rate of hippocampal atrophy metrics over time was conducted using mixed effects logistic regression models where the outcome variable was whether or not forward transition between disease states occurred since the previous visit. All models were adjusted for age, gender, education, APOE ε4 genotype, diagnosis at previous visit and time between visits. Models also included a random intercept to account for correlations between repeated measures within individuals. Four independent models were developed using different combinations of markers. Models 1 and 2 included HV_res_ at previous visit and HV_rate_ independently; model 3 included both metrics in the same model simultaneously; model 4 extended model 3 to include the interaction between both metrics. Models were compared using Bayesian Deviance Information Criterion (DIC). Odds ratios were also converted to probabilities of transitioning from state to state given a fixed set of covariates. In order to investigate whether the effect of HV metrics on the progression of AD differs depending on which stage of the disease process an individual is in (i.e. CN or MCI), interactions between HV metrics and previous diagnosis were included, as per the methodology described above.

The level of statistical significance used in simple comparisons and model output was set to be *p* < 0.05 and all tests were two-sided. All analyses were conducted using R statistical software^26^ primarily using packages *survival*^27^, *pROC*^28^, and *R2MLwiN*^29^ for the survival, ROC and mixed effects analyses, respectively.

## Results

### Characteristics of the sample

Demographics of the sample and summary statistics of HV metrics at baseline by diagnostic state can be found in Table 2. There was a significant difference in age at baseline between CN and MCI groups (p = 0.015), however, the effect size was small (Cohen’s D = 0.219). There was no significant difference in the distribution of education between diagnostic groups however significant differences in the distributions of gender and APOE ε4 genotype were observed (p = 0.002 and p < 0.001 respectively).

### Predictive value of HV metrics

**Figure 1:**
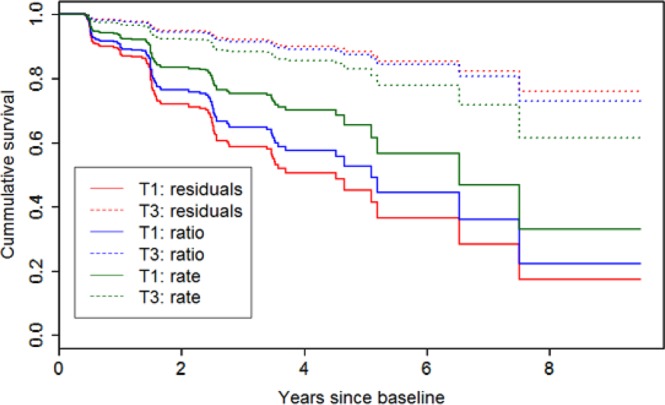
Kaplan-Meier estimates for cumulative survival related to AD events by tertile of HV metric at baseline. Abbreviations: T1 = Tertile 1; T3 = Tertile 3.

Lower values related to HV_res_, HV_ratio_ and HV_rate_ were associated with higher risk of developing AD for those diagnosed as MCI at baseline (Table 3). In all three cases, the HRs for HV_res_ were higher than those associated with HV_ratio_ and HV_rate_. Figure 1 presents the Kaplan-Meier estimates depicting cumulative survival for each metric by tertile at baseline.

**Table 3:** Number of AD-progressors and hazard ratios by HV metric and tertile. ^a^Hazard ratios were adjusted for age at baseline, gender, education and APOE ε4 genotype.

	Tertile	Non-AD cases	AD cases	Hazard Ratio^a^	95% CI
HV Residuals (HV_res_)	**T1**	81	14	ref	ref
	**T2**	49	45	3.96	(2.16, 7.26)*
	**T3**	40	55	6.42	(3.45, 11.95)*
HV Ratio (HV_ratio_)	**T1**	76	19	ref	ref
	**T2**	54	40	3.00	(1.71, 5.25)*
	**T3**	40	55	4.78	(2.74, 8.34)*
HV Rate (HV_rate_)	**T1**	71	24	ref	ref
	**T2**	54	40	2.06	(1.23, 3.44)*
	**T3**	45	50	2.28	(1.39, 3.74)*

*p < 0.001.

AUC values were calculated from the ROC curves that were fitted to continuous HV_res_, HV_ratio_, and HV_rate_ as measures of the predictive ability of each of these metrics at discriminating between disease states. AUC values for HV_res_ were 0.767, 0.751 and 0.722 for events limited to those that happened within one year, three years and five years from baseline, respectively. AUC values for HV_ratio_ for the same time intervals were 0.742, 0.732 and 0.707, whereas those for HV_rate_ were 0.673, 0.641 and 0.644. The ROC curves for HV_res_ and HV_ratio_ by follow-up time can be found as Supplementary Fig. S1.

As HV_res_ and HV_ratio_ are considered normalised metrics using different methodologies, only the metric with the better predictive value, in this case HV_res_, was carried forward and used in the longitudinal modelling component of this study.

### The effect of HV over time

In total, 561 individuals contributed 1,509 visits/diagnoses to the models presented below. Of the four models developed, model 3 (both HV_rate_ and HV_res_ main effects included but no interaction) fit the longitudinal data from ADNI best with the smallest deviance information criterion (DIC = 924). Differences in DIC between models ranging from 5 to 10 are considered to provide substantial evidence in favour of the model with the smallest DIC^30^. This suggests that there is value in considering HV and rates of hippocampal atrophy simultaneously when attempting to define disease progression. Model 4, which includes an interaction term between HV metrics, did not improve the fit relative to model 3. The non-significant interaction term and larger DIC suggests that the effect of each HV term is not influenced by the presence of the other. In other words, the effect of rate of hippocampal atrophy is constant for two individuals with different absolute HVs.

The odds ratios (see Table 4) associated with HV_res_ and HV_rate_ were 0.34 (95% CI = (0.25, 0.46)) and 0.53 (95% CI = (0.34, 0.83)) respectively, indicating that the smaller HV and larger rate of hippocampal atrophy were associated with increased likelihood of transitioning to a more severe disease state (i.e. disease progression). Also associated with an increased likelihood of transitioning was being APOE ε4 heterozygous relative to negative as well as an increased length of time between visits (odds ratios of 1.69 and 2.58 respectively).

**Table 4:** Output from the four generalised linear models (all of which include a fixed set of covariates plus combinations of HV metrics).

Fixed		Model 1 (DIC = 936)		Model 2 (DIC = 1,011)		Model 3 (DIC = 924)		Model 4 (DIC = 933)	
		OR (95% CI)	*p*	OR (95% CI)	*p*	OR (95% CI)	*p*	OR	*p*
Intercept		0.81 (0.06, 11.6)	*	0.03 (0.00, 0.29)	**	0.87 (0.06, 12.3)		1.02 (0.10, 10.6)	*
Time between Visits (year)		2.61 (1.85, 3.67)	***	2.09 (1.61, 2.72)	***	2.58 (1.82, 3.65)	***	2.47 (1.77, 3.43)	***
Age at previous visit		0.96 (0.93, 0.99)		1.01 (0.98, 1.04)		0.95 (0.92, 0.99)	**	0.95 (0.93, 0.98)	**
Gender (ref = male)	Female	0.85 (0.56, 1.30)		0.95 (0.67, 1.36)		0.83 (0.54, 1.30)		0.84 (0.55, 1.28)	
Education (year) (ref: ≤12)	>12	1.09 (0.62, 1.91)		1.09 (0.69, 1.73)		1.08 (0.61, 1.93)		1.10 (0.63, 1.92)	
APOE genotype (ref = −/−)	+/−	1.75 (1.1, 2.79)	*	1.83 (1.26, 2.66)	**	1.69 (1.03, 2.77)	*	1.65 (1.05, 2.61)	*
	+/+	1.80 (0.91, 3.56)		2.15 (1.25, 3.71)	**	1.67 (0.83, 3.35)		1.65 (0.87, 3.13)	
Diagnosis at previous visit	MCI	0.96 (0.56, 1.68)		1.95 (1.25, 3.04)	**	0.90 (0.5, 1.61)		0.90 (0.52, 1.56)	
HV_res_ at previous visit (cm^3^)		0.36 (0.27, 0.49)	***	—		0.34 (0.25, 0.46)	***	0.36 (0.26, 0.49)	***
HV_rate_ since last visit (cm^3^/year)		—		0.54 (0.37, 0.8)	**	0.53 (0.34, 0.83)	**	0.48 (0.24, 0.95)	*
HV_res_: HV_rate_ interaction		—		—		—		0.92 (0.60, 1.42)	
Random		Variance		Variance		Variance		Variance	
ID (Intercept)		0.845		<0.001		1.051		0.702	

Model 1: Base +  HV_res_; Model 2: Base + HV_rate_; Model 3: Base + HV_res_ + HV_rate_; Model 4: Base + HV_res_ *HV_rate_. Abbreviations: OR = Odds ratio; CI = Confidence interval; SD = Standard deviation; DIC = Deviance Information Criterion, *p ≤ 0.05; **p ≤ 0.01; ***p ≤  0.001; blank = not significant.

Interactions between HV_res_ and HV_rate_, and previous diagnosis were added to model 3 simultaneously to determine whether the effect of each metric on the progression of AD differed depending on whether individuals were diagnosed as CN or MCI. Neither interaction was significant indicating that there is insufficient evidence to say that the effects of HV and rate of hippocampal atrophy vary by diagnosis. In other words, the effect of these metrics is constant over the preclinical continuum of the disease i.e. CN and MCI.

**Figure 2:**
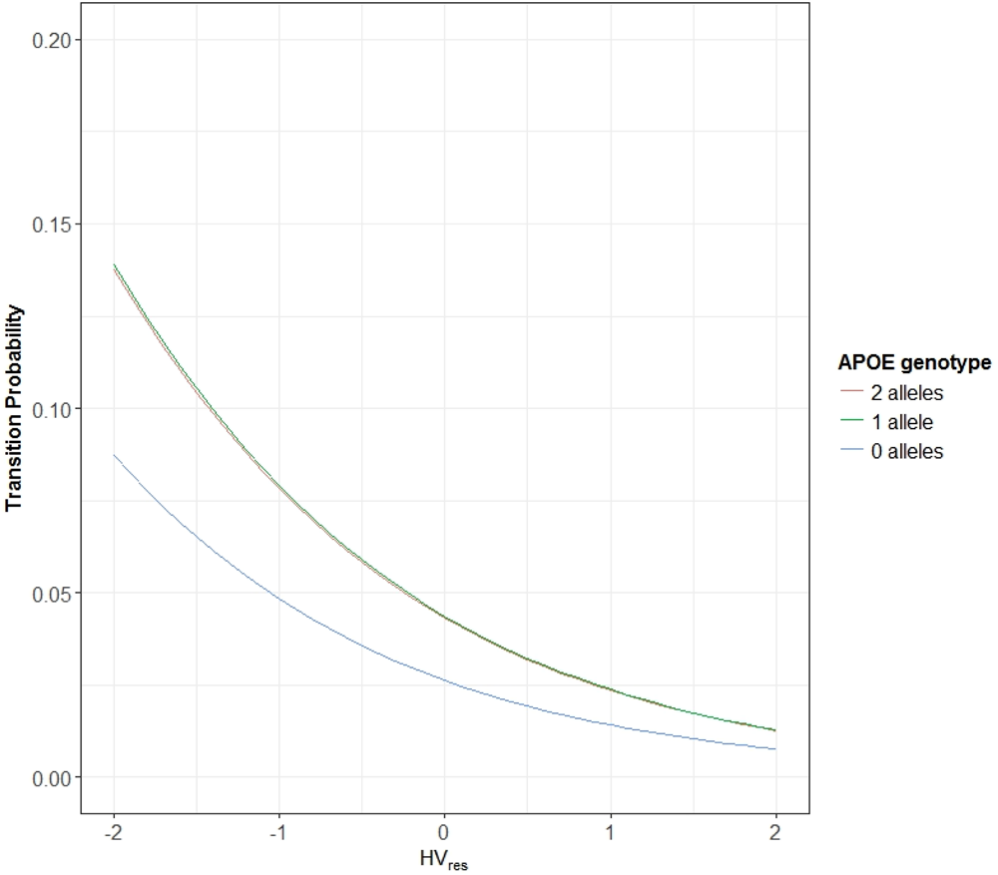
Estimated probabilities of transitioning to AD (from MCI at previous visit) for varying levels of HV_res_ by APOE ε4 genotype derived using Model 3. Probabilities represent those that are male, have at most 12 years of education, and have age and HV_rate_ fixed at their respective sample means.

The probabilities of transitioning for a given set of fixed covariates can be estimated using the odds ratios presented in Table 4. For example, Fig. 2 illustrates the estimated probabilities of transitioning for varying levels of HV_res_ across different APOE ε4 genotypes. APOE ε4 genotype was chosen for visualisation purposes; transition probabilities can be calculated across any combinations of variables depending on the characteristics of the sample.

## Discussion

Extremely high failure rates for recent clinical trials of possible AD therapies may be associated with end point choice, recruitment strategies and large variation in both outcome measures and markers of disease^4^. This motivates the need for better AD biomarkers that may be used to identify individuals that are more likely to develop disease and thereby potentially demonstrate faster cognitive decline in a clinical trial. This study builds upon recently implemented inclusion criteria that may restrict the study population to at-risk individuals (e.g. individuals with high levels of amyloid measured in CSF or PET) based on single measurements at an arbitrary point in time.

With consistently high AUC values across studies, HV has been shown to be a good predictor for conversion to AD thus providing a useful tool for improving the design of clinical trials of potential AD therapies. This analysis focused on the association between HV and AD progression over time in a sample of at-risk individuals with the objective of determining whether it is preferable to use a combination of HV metrics over any single metric alone.

We began by cross-validating previous findings by showing high HR and AUC values for the association of HV metrics and transitions from MCI to AD. We then showed, utilising repeated measurements within individuals over time, that a combination of absolute HV and rate of hippocampal atrophy is preferred to either metric on its own when trying to understand transitions to more severe states using longitudinal MRI, clinical and demographic data from the ADNI. The estimation of the probabilities of transitioning between different clinical states has been shown to provide further insights into the potential reasons why clinical trials of potential AD therapies have failed^17,31^. This result is in line with that from other cross-sectional studies that suggest that combinations of HV metrics improve the predictive accuracy of HV in general^12^.

Whilst differences in absolute HV are informative at the population level, differences in rates of hippocampal atrophy are required in order to understand transitions between states at the individual level. This is particularly important in the absence of well-defined individual-level trajectories of HV over long periods of time. Combining HV metrics in a way allows us to consider where on a hypothetical HV trajectories an individual may lie at a given point in time^32^.

The absence of a significant interaction between HV_res_ and HV_rate_ indicates that although there is added benefit in including both metrics simultaneously, the effect of the rate of hippocampal atrophy does not vary for different measures of absolute HV and vice versa. The odds ratios estimated using the generalised linear mixed models showed that smaller absolute HV and larger rate of hippocampal atrophy were both associated with increased likelihoods of transitioning to a more severe disease state. This builds on other studies in the literature^12^ by extending the result of combinations of metrics on a binary outcome (e.g. AD versus non-AD progressors) to the more gradual nature of AD progression, from CN to MCI to AD. The shift to a more granular transition model for the progression of AD will be crucial as clinical trials of potential preventative or disease-delaying therapies of AD are aimed more towards individuals in the preclinical stages of AD as opposed to prodromal stages^7^.

When the model was extended to include an interaction between each HV metric and diagnosis, the lack of significance associated with this term in all cases suggested that the effects of HV metrics did not differ significantly between diagnoses of CN and MCI. In other words, the effect of these metrics may be constant over the preclinical continuum of the disease.

One limitation of this study, despite its current use in practice, is that a strict binary cut point was fixed to define amyloid abnormality. It is important to note that dichotomising a continuous measure may lead to misclassification, especially for individuals near the cut point, and is sensitive to longitudinal changes in the measure over time, given that the measurement is taken at some arbitrary point time (e.g. study entry). That being said, the purpose of limiting this study to amyloid positive individuals was in part to place this analysis in the context of recent clinical trials.

Logical extensions to this study include (1) the quantification of the effect of HV metrics on other outcome measures, such as cognitive and functional scores, (2) the consideration of the magnitude and impact of measurement error within these models and (3) similar analysis of combinations of other AD related markers, for example, FDG or levels of t-tau or Aβ_42_ in plasma and/or CSF.

Firstly, the endpoints chosen for phase III clinical trials for potential AD therapies are related to cognition or activities of daily living. For example, recent trials^2^ used the Alzheimer’s Disease Assessment Scale – Cognitive Subscale (ADAS-Cog), Clinical Dementia Rating Scale – Sum of Boxes (CDR-SB) and Alzheimer’s Disease Cooperative Study – Activities of Daily Living (ADCS-ADL). Although modelling transitions between clinically diagnosed states should capture some of the variation in these scores, it would be interesting to quantify the effect of these HV metrics on cognitive and functional scores directly. Future work would extend the models presented here to explore the relationship between the HV metrics and cognition, as opposed to using clinical states.

Secondly, it is evident that the consideration of the rate of hippocampal atrophy over time introduces additional variation both between and within individuals over time (see Supplementary Fig. S2). This added noise may either be due to true variation in the rate of hippocampal atrophy within individuals over time or due to measurement error introduced at either the acquisition or image processing stages. The literature related to the magnitude and impact of this measurement error is as yet limited. However, it is important to note that analyses that do not account for measurement error in some way will be subject to certain levels of attenuation in terms of the effect of HV on the outcome^33^. Further work is required in this area to fully understand the amount and effect of measurement error on the outcomes of interest.

Lastly, due to the increase in the frequency of collection of other imaging markers (e.g. FDG and PiB) and the development of assays to improve the detection of biomarkers (e.g. t-tau or Aβ_42_) in plasma over recent years, one logical next step should be to conduct similar longitudinal analysis of these markers. Furthermore, longitudinal analysis of the effect of these markers on the progression of AD would provide valuable insights into the timing of amyloidosis and neurodegeneration relative to disease onset during the preclinical phases of AD in particular. Emphasis should also be placed upon developing the trajectories of these markers over time^34^. In the long term, these AD-related markers may be shown to be preferred endpoints in clinical trials.

## Conclusions

The findings from this study indicate that HV and rate of hippocampal atrophy should be used in tandem when describing AD progression in at-risk individuals. The effects of smaller HV and higher rate of hippocampal atrophy were to increase the likelihood of transitioning to a more severe disease state, hence could be utilised during the recruitment phase of clinical trials. Patients that progress towards AD at a faster rate should demonstrate larger changes in outcome measures over time e.g. cognitive of functional score, hence increasing the likelihood of detecting a significant treatment effect.

## Supplementary information

Supplementary Information

## Data Availability

Data used in preparation of this article were obtained from the Alzheimer’s Disease Neuroimaging Initiative (ADNI) database (adni.loni.usc.edu). The primary goal of ADNI has been to test whether serial MRI, PET, other biological markers, and clinical and neuropsychological assessment can be combined to measure the progression of mild cognitive impairment and early Alzheimer’s disease. The Principal Investigator of ADNI is Michael W. Weiner, MD (email: Michael.Weiner@ucsf.edu).
