# Supplementary material for: Combining hippocampal volume metrics to better understand Alzheimer’s disease progression in at-risk individuals
Source: Sci Rep. 2019 May 16;9:7499. doi: 10.1038/s41598-019-42632-w (PMC6522521; doi:10.1038/s41598-019-42632-w)
Supplement: Supplementary file 1 — Supplementary Information [file 41598_2019_42632_MOESM1_ESM.pdf]

Title Page

Combining hippocampal volume metrics to better understand Alzheimer's disease progression in at-risk individuals

McRae-McKee K.[1, *], Evans, S.[1], Hadjichrysanthou, C.[1], Wong, MM.[1], de Wolf, F.[1, 2], and Anderson, RM.[1], for the Alzheimer's Disease Neuroimaging Initiative

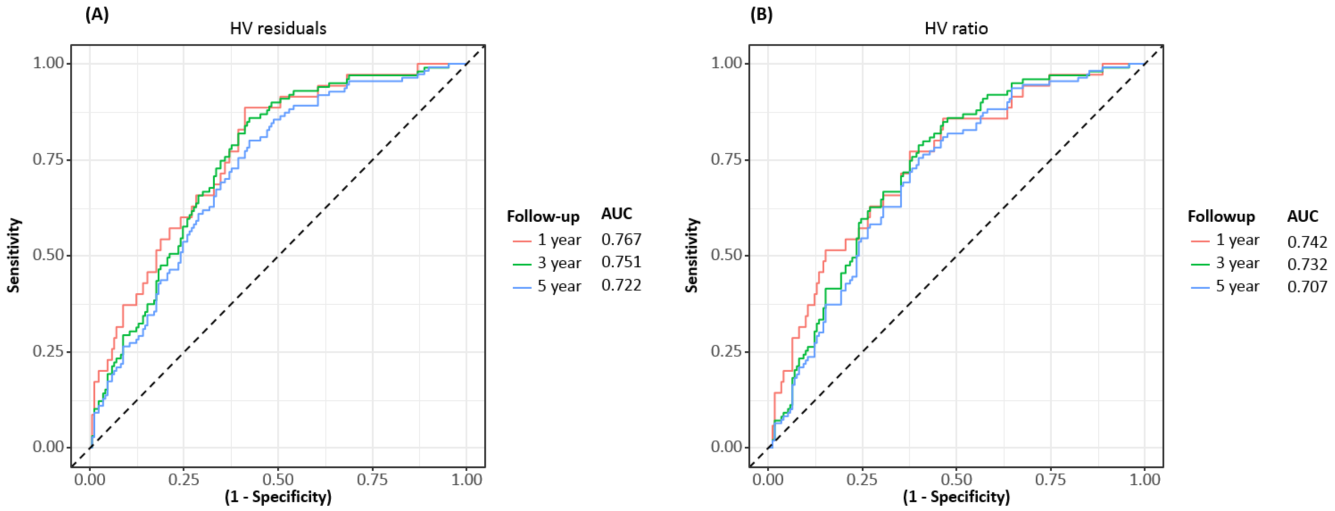

Supplementary Figure S1: ROC curves for (A) HV_{res} and (B) HV_{ratio} with associated AUC estimates. The red, green and blue curves represent events within 1, 3 and 5 years respectively. Abbreviations: AUC = Area Under Curve.

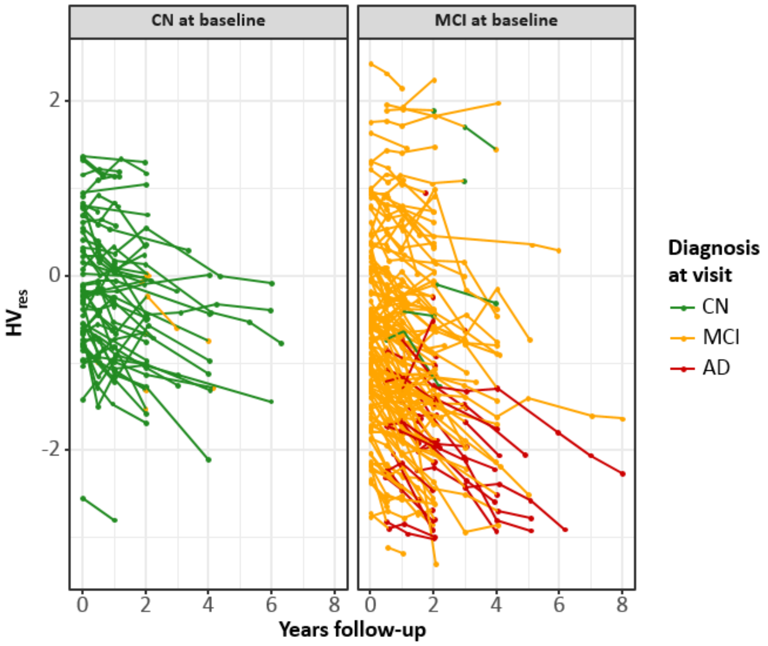

Supplementary Figure S2: HV_{res} over time for a random sample of individuals (200/561). Separate figures represents baseline diagnoses of either CN or MCI whereas colours within figure represent the diagnosis at each individual visit over time.
